# Induction and Prolonged Induction With Mirikizumab in Ulcerative Colitis—A Prospective, Real‐World Study From the Sicilian Network for Inflammatory Bowel Disease (SN‐IBD)

**DOI:** 10.1002/ueg2.70244

**Published:** 2026-06-20

**Authors:** Giuseppe Costantino, Anna Viola, Andrea Iuculano, Fabio Salvatore Macaluso, Raffaele Li Voti, Maria Cappello, Chiara Antoci, Concetta Ferracane, Morena Sciuto, Domenico Catarella, Stefanie Parisi, Filippo Mocciaro, Barbara Scrivo, Maria Emanuela Distefano, Roberto Vassallo, Maria Giovanna Minissale, Giuseppe Scalisi, Serena Garufi, Sofia Rao, Stefano Muscarella, Vincenza Tortorella, Emiliano Giangreco, Marco Muscianisi, Ambrogio Orlando, Walter Fries

**Affiliations:** ^1^ IBD Unit Department of Clinical and Experimental Medicine University of Messina Messina Italy; ^2^ IBD Unit Villa Sofia‐Cervello Hospital Palermo Italy; ^3^ Gastroenterology and Hepatology Section PROMISE University of Palermo Palermo Italy; ^4^ Gastroenterology Unit AOU Policlinico San Marco Catania Italy; ^5^ Gastroenterology Unit Garibaldi‐Nesima Hospital Catania Italy; ^6^ Gastroenterology and Endoscopy Unit ARNAS Civico‐Di Cristina‐Benfratelli Hospital Palermo Italy; ^7^ IBD Unit Cannizzaro Hospital Catania Italy; ^8^ Gastroenterology and Endoscopy Unit A.O. Buccheri la Ferla Fatebenefratelli Palermo Italy; ^9^ Gastroenterology Unit ASP CT—PO Paternò Paternò Italy; ^10^ Gastroenterology Unit S Antonio Abate Hospital Erice Italy; ^11^ Gastroenterology Unit A.O.O.R. Papardo Piemonte Messina Italy; ^12^ Gastroenterology Unit Guzzardi Hospital Vittoria Italy

**Keywords:** anti IL‐23 p19 agents, biological therapies, bowel urgency, effectiveness, ulcerative colitis

## Abstract

**Background:**

Mirikizumab (MIRI), an IgG4 monoclonal antibody targeting IL‐23 p19, was recently licensed for ulcerative colitis (UC). Most available data come from the pivotal trials, but real‐life data are scant. We aimed to evaluate the effectiveness and safety of MIRI in a real‐world setting.

**Methods:**

UC patients from 12 centres of the SN‐IBD were prospectively enrolled. The primary endpoints were clinical response, steroid‐free remission (SFR), and reduction of urgency at week 12 and at week 24. As secondary endpoint, the need for prolonged induction was considered. All patients received 3 infusions at monthly intervals of 300 mg MIRI; in case of inadequate response, the induction period was prolonged. Urgency was determined by means of the Urgency Numerical Rating Scale (UNRS).

**Results:**

One hundred and five patients were included. Previous failures to 2 or more lines of advanced therapies were present in 74% of patients. At week 12, 24% of patients achieved a clinical response and 53% achieved SFR. At week 24 (*n* = 71), the rates were 20% and 68%, respectively. The median urgency score decreased from 6 at baseline to 2 at week 12 and to 1 at week 24 (*p* < 0.001, both). Prolonged induction was necessary to half of patients. Adverse events were reported in 5% of patients. Treatment failures occurred in 14 patients (13%).

**Conclusions:**

Our real‐life study confirmed the short‐term effectiveness and safety of MIRI in patients with UC especially in relieving bowel urgency. The flexible induction schedule allows an additional gain in response and remission.

## Key Summary

- Summarise the established knowledge on this subject
  ◦ Mirikizumab is a monoclonal antibody targeting IL‐23 p19 that was recently approved for the treatment of ulcerative colitis.
  ◦ While mirikizumab has demonstrated efficacy in inducing and maintaining remission in clinical trials, real‐world evidence remains limited.
- What are the significant and/or new findings of this study?
  ◦ We prospectively enrolled UC patients starting Mirikizumab. The primary endpoints were clinical response, steroid‐free remission, and reduction of urgency at week 12 and at week 24. As secondary endpoint, the need for prolonged induction was considered.
  ◦ In a real‐world, highly refractory UC population, mirikizumab proved to be effective and safe with a marked improvement in bowel urgency.
  ◦ A flexible, prolonged induction strategy provided additional benefits, enhancing clinical response and remission rates.

## Graphical abstract

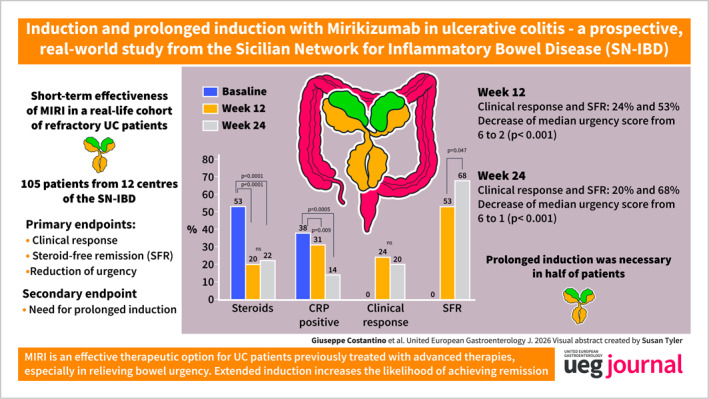

## 1 Introduction

Ulcerative colitis (UC) is a chronic inflammatory condition of the large intestine and rectum. Conventional treatments for mild‐to‐moderate disease, such as mesalamine, thiopurines, and corticosteroids, may be insufficient to control disease activity. Moderate‐to‐severe UC often requires biological therapies, which have become the mainstay of treatment for inflammatory bowel disease (IBD) refractory to conventional therapy or presents with severe activity. Over the past decade, the number of advanced therapies with different mechanisms of action has grown substantially. Biologics approved for UC include the anti–TNFα agents infliximab, adalimumab, and golimumab [1, 2, 3], the anti‐integrin vedolizumab [4], ustekinumab [5], an anti‐p40 antibody targeting the subunit shared by interleukin (IL)‐12 and IL‐23, and small molecules such as Janus kinase inhibitors (tofacitinib, filgotinib, and upadacitinib) and sphingosine‐1‐phosphate receptor modulators (ozanimod and etrasimod) [6, 7, 8, 9, 10].

One of the most recently identified pathways involved in UC pathogenesis is the IL‐23 pathway for which ustekinumab (USTE) was the first approved agent [5]. IL‐23 consists of two subunits, p40 and p19, the latter being unique to IL‐23 and offering a selective target for novel therapies. Mirikizumab (MIRI) (Omvoh, Lilly) is the first p19‐specific anti–IL‐23 monoclonal antibody approved for the treatment of UC. Data from the LUCENT clinical program demonstrated that MIRI is effective in inducing and maintaining clinical remission in patients with moderately to severely active UC, with an excellent safety profile [11]. In addition, MIRI significantly reduced bowel urgency compared with placebo, addressing one of the most burdensome symptoms reported by patients with UC. These findings require confirmation in real‐world cohorts to better define the therapeutic positioning of this biologics, including its potential role in difficult‐to‐treat or refractory patient populations.

We aimed to evaluate the short‐term effectiveness of MIRI in a real‐life cohort of refractory UC patients with a specific focus on steroid‐free remission (SFR) and improvement in bowel urgency. We also assessed treatment outcomes in patients previously exposed to USTE, a population excluded from the LUCENT trials.

## 2 Methods

### 2.1 Patients

In this multicentre prospective study including 12 Sicilian centres from SN‐IBD, data from patients with a confirmed clinical, endoscopic, and histological diagnosis of UC starting MIRI were collected between February 2025 and October 2025. Inclusion criteria were age ≥ 18 years and the ability to understand and provide informed consent. The only exclusion criteria were incomplete uploaded data or patients with less than 3 infusions. Patients who discontinued treatment before week 12 due to adverse events were included in the analysis. MIRI was prescribed according to the results of the LUCENT trial, which demonstrated efficacy and safety in a moderate‐to‐severe refractory UC population. Induction included intravenous administration of MIRI 300 mg every 4 weeks for the first 12 weeks, followed by subcutaneous administration of 200 mg every 4 weeks. Patients with incomplete response, persistent CRP elevation, or not weaned off steroids at week 12 could receive an additional three doses of intravenous MIRI over 12 weeks (extended induction) according to the treating physician's decision. The study was approved by the EC of the coordinating centre (Messina) with protocol 36/25, as of February 3, 2025, and data were collected and handled anonymously in compliance with European data protection laws.

### 2.2 Data Collection and Outcome Measures

The following data were collected for each patient in a common database: gender, age at diagnosis, age at therapy initiation, smoking status (never, former, or current), disease duration, partial Mayo score (pMS) at baseline, concomitant therapies, and comorbidities. Disease extent was classified according to the Montreal classification. Data on previous biological therapies, including the number and type of advanced agents, were also recorded. Comorbidities were assessed using the Charlson Comorbidity Index (CCI) [12]. Follow‐up data at week 12 and week 24 included clinical response according to the partial Mayo score (pMS), steroid‐free remission (SFR), and laboratory data including C‐reactive protein (CRP) and faecal calprotectin (Fcal). Due to variability of available kits used in local laboratories, data on Fcal were categorized as normal or elevated at baseline and as elevated, normalized, or declining in follow‐up (declining was defined as a reduction by 50% compared to baseline).

The wide variability of available kits used in local laboratories made numerical comparison difficult; thus, available data were categorized as normal or normalized, elevated, or declining.

All adverse events (AEs) and reasons for treatment discontinuation were recorded. Bowel urgency was evaluated using the Urgency Numerical Rating Scale (UNRS) [13], licensed under Creative Commons Attribution‐NoDerivatives 4.0 International. The UNRS is a validated tool ranging from 0 (no urgency) to 10 (worst possible urgency) with a reduction of 2 points representing a clinically significant improvement [14].

The primary endpoints were clinical response, steroid‐free remission, and reduction of bowel urgency. The secondary endpoint was the need for prolonged induction. Clinical response was defined as a reduction in pMS ≥ 3 points from baseline. Steroid‐free remission was defined as a 9‐point pMS < 2 without concomitant steroids [15]. There was no standardized steroid tapering regimen.

Primary treatment failure was defined as persistent symptoms and serologic evidence of inflammation at the end of induction, whereas loss of response was defined as worsening of symptoms and serologic inflammation after an initial response. Treatment persistence was assessed at weeks 12 and 24.

### 2.3 Comparison Between Patients Naïve or Previously Exposed to Anti‐TNFs, USTE, VEDO, or JAKi

To evaluate whether prior treatment with anti‐TNFs, USTE, VEDO, or JAKi influenced outcomes, patients were divided into groups based on previous exposure to former advanced therapies using the same outcome measures as in the overall cohort.

### 2.4 Statistics

Statistical analyses were performed using SPSS version 22.0 for Windows. Categorical variables were reported as absolute frequencies and percentages. Continuous variables were reported as mean ± standard deviation (SD) or median with interquartile range (IQR). Univariate and multivariate logistic regression analyses were conducted to identify predictors of outcome. Changes in clinical scores (pMS, UNRS) between baseline and week 12 were evaluated using the Wilcoxon signed‐rank test. Differences across baseline, week 12, and week 24 were analyzed using the Friedman test for repeated measures, limited to patients with complete data. Changes in proportions over time were evaluated using McNemar's test. Kaplan‐Meier survival analysis was used to assess treatment persistence, considering the time from initiation to week 12 or 24, or treatment discontinuation. Efficacy outcomes were primarily analyzed using a per‐protocol approach, including patients with available follow‐up at each time point. An additional intention‐to‐treat (ITT) analysis including the entire cohort was performed as a sensitivity analysis with missing data handled by non‐responder imputation.

### 2.5 Sample Size Calculation

Based on efficacy data from the Phase 3 LUCENT trial [11], where mirikizumab achieved a clinical remission rate of approximately 24% at week 12, we assumed a similar expected remission rate in our real‐world cohort. Considering a precision of ± 10% with a 95% confidence level, the estimated minimum sample size was approximately 70 patients. Accounting for an anticipated dropout rate of about 15%, at least 80 evaluable patients were required. Given the multicentre real‐world design and the recruitment capability of the participating centres, the planned enrolment target was set at approximately 100 patients.

## 3 Results

### 3.1 Patients and Baseline Characteristics

Data from 105 patients initiating MIRI were prospectively collected. Baseline characteristics are summarized in Table 1. Mean age at MIRI initiation was 51 ± 16 years, 45/105 (43%) were female, and 8/105 (8%) were current smokers. The median disease duration was 9 years. Almost all patients 104/105 (99%) had received at least one prior biologics, and 78/105(74%) were multi‐failure to more than one advanced therapy. Only one patient was naïve to advanced therapies. At baseline, median pMS was 5 points, 56/105 (53%) of patients were on steroids, and CRP was elevated in 45/105 (40%). Sixteen patients (15%) had concomitant extraintestinal manifestations (EIMs).

**TABLE 1:** Baseline characteristics of patients.

	*N* = 105
Gender (female); *n*/*N* (%)	45/105 (43)
Age (yrs) mean ± SD	51 ± 16
Active smokers; *n*/*N* (%)	8/105 (8)
Ex smokers; *n*/*N* (%)	28/105 (27)
IBD duration (yrs); median (range)	9 (1–45)
Montreal UC; *n*/*N* (%)	
E1; *n*/*N* (%)	5/105 (5)
E2; *n*/*N* (%)	60/105 (57)
E3; *n*/*N* (%)	40/105 (38)
Partial mayo score; median (range)	5 (0–9)
Endoscopic mayo score; median (range)	2 (1–3)
CRP positive at baseline; *n*/*N* (%)	40/105 (38)
Faecal calprotectin elevated; *n* (%)	75/87 (86)
(Missing = 18)	
Steroids at baseline; *n*/*N* (%)	56/105 (53)
Steroid dependency; *n*/*N* (%)	60/105 (57)
Steroid resistance; *n*/*N* (%)	5/105 (5)
Previous biologics; *n*/*N* (%)	104/105 (99)
Anti‐TNFs; *n*/*N* (%)	88/105 (84)
Vedolizumab; *n*/*N* (%)	46/105 (44)
Ustekinumab; *n*/*N* (%)	36/105 (34)
Anti‐JAKs; *n*/*N* (%)	41/105 (39)
Failure to 1 advanced therapy; *n*/*N* (%)	26/105 (25)
Failure to 2 advanced therapy; *n*/*N* (%)	30/105 (28)
Failure to 3 advanced therapy; *n*/*N* (%)	48/105 (46)
Extended induction; *n*/*N* (%)	57/105 (54)
EIMs; *n*/*N* (%)	16/105 (15)

Abbreviations: CRP = C‐reactive protein; EIMs = extra intestinal manifestations; JAK = Janus kinase, SD = standard deviation.

Endoscopic activity at baseline was moderate with a median Mayo endoscopic subscore of 2 (range 1–3). Faecal calprotectin at baseline was available in 87 (83%) patients and was elevated in 75/87 (86%) patients.

### 3.2 Treatment Effectiveness and Predictors of Failure

#### 3.2.1 Week 12 Outcomes

**FIGURE 1:**
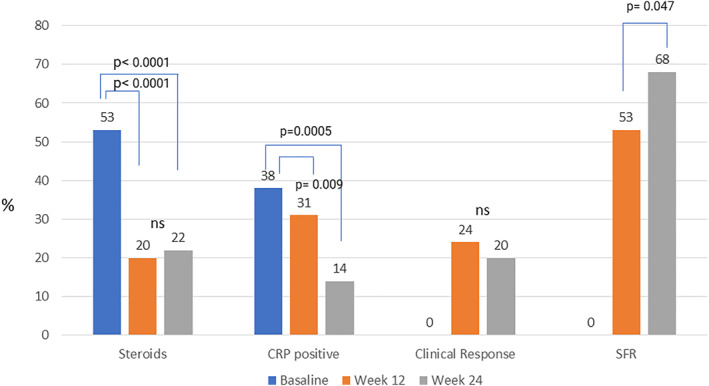
Effectiveness of mirikizumab at weeks 12 and 24 compared to baseline (per protocol analysis). CRP = C‐reactive protein; SFR = steroid‐free remission.

**FIGURE 2:**
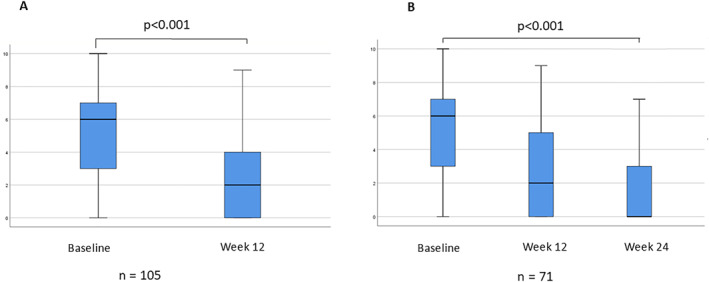
Reduction in bowel urgency from baseline to week 12 (A) and from baseline to week 24 (B).

At week 12, clinical response and SFR were achieved in 24% and 53% of patients, respectively. Persistent CRP elevation was observed in 31%, and 21 patients (20%) remained on steroids (Figure 1). Mean pMS decreased significantly from 4.95 ± 2.3 to 2.24 ± 2.2 (*p* < 0.001). Median UNRS also decreased significantly (*p* < 0.001; Figure 2A). Treatment persistence at week 12 was 93.3% (Figure S1A). Fcal was available from 52 patients (50%) at week 12: it remained elevated in 12/52 (23%), normalized in 12/52 (23%), and declining in 28/52 (54%).

#### 3.2.2 Week 24 Outcomes

Data from 71 patients reaching week 24 were available. In these patients, clinical response and SFR were 20% and 68%, respectively (Figure 1). ITT analysis showed 14% of clinical response and 53% of SFR (data not shown). Mean pMS decreased from 4.95 ± 2.4 at baseline to 1.8 ± 2.4 at week 24 (*p* < 0.001). UNRS values also decreased significantly (*p* < 0.001; Figure 2B). Extended induction was required in 57/105 patients (54%), with complete week 24 data available for 43 patients. Among these, SFR was achieved in 22/43 (51%) and clinical response in 13/43 (30%). Overall treatment persistence at week 24 was 89.5% (Figure S1B). At week 24, data on Fcal were available in 36/71 patients (51%) at week 24: it remained elevated in 9/36 (25%), normalized in 19/36 (53%), and declining in 8/36 (22%).

### 3.3 Safety and Treatment Discontinuation

During follow‐up, 17/105 patients (16%) discontinued MIRI: 14 (13%) due to treatment failure and 3 due to AEs (2 within week 12, 1 within week 24). AE‐related discontinuations included oral cavity lesion (*n* = 1), bronchospasm (*n* = 1), and proctocolectomy (*n* = 1). Among treatment failures, 5 occurred within the first 12 weeks, and 9 occurred during the subsequent 3‐month period. These patients continued to have active disease (mean pMS 6.5) and UNRS ≥ 5; seven had received extended induction. Univariate regression analysis including gender, disease duration and extent, baseline steroid use, steroid dependency, failure to > 2 ot > 3 advanced therapies, severe endoscopic activity at baseline, prior USTE exposure, and CRP positivity did not identify any significant predictors of treatment failure (Table 2).

**TABLE 2:** Univariate logistic regression.

Predictors	Treatment failure		
	OR	CI	*p*
Male gender	0.932	0.235–3.688	0.920
Disease duration^a^	0.643	0.152–2.722	0.549
Pancolitis	0.509	0.098–2.654	0.423
Steroid dependency	0.571	0.144–2.262	0.425
Steroids at baseline	8.000	0.963–66.45	0.054
Previous ustekinumab	1.600	0.402–6.370	0.505
Failure to 1 biologic	0.355	0.042–2.982	0.340
Failure to 2 biologics	0.094	0.158–4.167	0.802
Failure to > 2 biologics	1.541	0.390–6.093	0.538
CRP positive at baseline	0.183	0.022–1.520	0.116
Endoscopic Mayo score ≥ 2	1.041	0.201–5.375	0.962
Endoscopic Mayo score = 3	0.961	0.186–4.964	0.962

Abbreviations: CI = confidence interval; CRP = C‐Reactive Protein; OR = odds ratio.

^a^ More than 10 years.

### 3.4 Treatment Outcomes According to Prior Advanced Therapy Exposure

Baseline differences were observed across subgroups according to prior treatment exposure. USTE‐experienced patients were older (*p* = 0.002) and had received a higher number of previous advanced therapies (*p* = 0.005) compared with USTE‐naïve patients (Table S1A). The need for extended induction did not differ between the groups. At week 12, USTE‐naïve patients showed a borderline greater reduction in bowel urgency (*p* = 0.047), although this difference was not maintained at week 24. No significant differences were observed in pMS (week 12: *p* = 0.55; week 24: *p* = 0.26), clinical response (week 12: *p* = 0.74; week 24: *p* = 0.47), or SFR (week 12: *p* = 0.39; week 24: *p* = 0.93). Rates of AEs and treatment failures were also similar between the groups (Table S2A). Similar results were observed in the ITT analysis (Table S3A).

According to baseline characteristics, anti‐TNF–naïve patients were older (*p* < 0.001), had received fewer prior advanced therapies (*p* = 0.01), and were less frequently treated with steroids at baseline (*p* = 0.05) (Table S1B). The need for extended induction did not differ between the groups. At week 12, anti‐TNF–naïve patients showed a higher rate of AEs (*p* = 0.04), although this difference was not observed at week 24. No significant differences in efficacy outcomes were observed at either timepoint (Table S2B). Similar findings were reported in the ITT analysis (Table S3B).

Regarding prior VDZ exposure, demographic characteristics did not differ between VDZ‐experienced and VDZ‐naïve patients (Table S1C). VDZ‐naïve patients had received fewer prior advanced therapies (*p* < 0.001) but were more frequently on steroids at baseline (*p* = 0.001), while VDZ‐experienced patients showed higher baseline CRP levels (*p* = 0.01). The need for extended induction did not differ between the groups. No significant differences were observed in efficacy or safety outcomes at either timepoint (Table S2C) except for a higher rate of bowel urgency at week 12 in VDZ‐experienced patients, which was not maintained at week 24. Similar results were reported in the ITT analysis (Table S3C).

Finally, demographic characteristics did not differ between anti‐JAK–experienced and JAKi‐naïve patients (Table S1D), although JAKi‐naïve patients had received fewer prior advanced therapies (*p* < 0.001). No significant differences were observed in efficacy or safety outcomes at either timepoint (Table S2D). The only significant finding emerged in the ITT analysis at week 24, where a higher rate of SFR was observed in JAKi‐naïve patients (*p* = 0.02) (Table S3D).

## 4 Discussion

This is the first prospective real‐world study evaluating MIRI in a UC cohort beyond the standard 12‐week induction, including assessment of bowel urgency.

In the Lucent‐1 trial, 12‐week overall response and clinical remission rates were 63.5% and 24.2%, respectively, with higher remission in biologic‐naïve patients (30.9%) [16]. In our cohort, 104/105 patients had prior advanced therapy exposure, yet SFR was achieved in 53%. Takagi et al. reported 12‐week clinical remission of 44.2% [17]. Compared to their study, a higher proportion of our patients were on steroids at baseline (53% vs. 40.4%) and the percentage of CRP‐positive patients was lower (38% vs. 57.7%), possibly reflecting a lower inflammatory burden. In that study, 30.8% of patients were on concomitant tacrolimus (30.8%) or thiopurines (25%).

Case series in patients with high rates of secondary USTE failure reported 12‐week remission rates of 70% [18], and a small French study reported 83% [19], whereas two recent retrospective study reported 16% SFR and 43% at 12 weeks [20, 21].

This variability likely reflects differences in methodology, baseline characteristics, and sample sizes.

Unlike Lucent‐2 [22], where non‐responders to 12 week induction received additional intravenous MIRI, our extended induction decisions were individualized based on incomplete clinical response, ongoing steroid use, or elevated CRP. Overall, 54% of our patients underwent extended induction. Among 43 patients with extended induction and complete week 24 data, SFR increased by 51%, emphasizing the value of flexible induction schedules.

Bowel urgency decreased from a median UNRS of 6 at baseline to 2 at week 12 and to 1 at week 24. Although not included in the pMS, urgency is a major contributor to reduced quality of life. We used the self‐administered UNRS developed by Dubinsky et al. [14], which was also used in the pivotal trials. Urgency is often not considered by treating physicians and is underreported due to patient embarrassment. Currently, the American Food and Drug Administration (FDA) and the European Medicines Agency (EMA) do not consider urgency improvement as a primary endpoint in IBD drug trials [23]. Reductions in urgency likely parallel decreases in clinical activity and are not unique to MIRI [24]. Urgency may persist independently of inflammation in long‐standing disease due to a reduced bowel function [25, 26].

Comparison of study subgroups according to former treatments with advanced therapies did not reveal important changes in outcome. The number of anti‐TNF‐ naïve was very low, as in former reports [17, 20]; USTE‐experienced versus USTE‐naïve patients revealed no major differences in treatment success despite an older age and more prior therapy‐lines among USTE‐experienced patients. The significant earlier reduction in urgency at week 12 in USTE‐naïve patients was not confirmed at week 24. Similarly, prior treatments with VEDO or Jaki did not influence clinical outcomes at week 24, with one exception concerning the percentage of SFR in JAKi‐naïve patients.

Treatment failure in the first 12 weeks occurred in 5%, similar to other real‐world studies (5.4% [20], 8% [17], and 3% [21]). These data may be underestimated due to the possibility of extending induction. At study end, 13% were classified as treatment failures. These patients showed at week 24 still a high UNRS (score 5–9), elevated CRP, and inability to taper steroids.

No predictors of MIRI failure, including prior USTE exposure, were identified. Safety was consistent with previous studies with only three AE‐related discontinuations.

Strengths of this study include its prospective design, sample size, and 24‐week follow‐up. Limitations include lack of endoscopic evaluation at 24 weeks and the only partial availability of Fcal measurements. Since the latter parameter is not included in the list of analyses covered by the cost exemptions code, thus representing additional costs for the patients, collection of this data was not mandatory for the participating centres.

In conclusion, MIRI is an effective therapeutic option for UC patients previously treated with advanced therapies, including USTE. Extended induction increases the likelihood of achieving remission but may contribute to overestimation of drug persistence at least in the first trimester. Longer‐term studies are needed to confirm treatment persistence particularly in patients requiring prolonged induction.

## Author Contributions

G.C., A.V. and W.F. conceived and designed the study and drafted the manuscript. All other authors collected data and critically revised the manuscript for important intellectual content and approved the final version of the manuscript for submission.

## Funding

The authors have nothing to report.

## Conflicts of Interest

G.C. Advisory board/Lectures for Johnson & Johnson, Galapagos, Abbvie. A.V. Advisory board/Lectures for Pfizer, Johnson & Johnson, Galapagos, Takeda, Eli‐Lilly. F.S.M served as an advisory board member and/or received lecture grants from AbbVie, Alfasigma, Cadigroup, Galapagos, Giuliani, Janssen, Lilly, Lionhealth s.r.l., Pfizer, Takeda Pharmaceuticals, Sandoz. M.C. has served as invited speaker and advisory board member for Janssen, Takeda, Pfizer, AbbVie, Sandoz, Ferring, Eli Lilly, Galapagos. F.M. Advisory board/Lectures for AbbVie, Alfasigma, Galapagos, Takeda, Eli‐Lill, y, Johnson & Johnson, Sandoz, Ferring. B.S. Advisory board/Lectures for Takeda. A.O. served as an advisory board member and/or received lecture grants from AbbVie, Alfasigma, Cadigroup, Galapagos, Ferring, Fresenius Kabi, Giuliani, Janssen, Lilly, Lionhealth s.r.l., MSD, Pfizer, Sandoz, Takeda Pharmaceuticals. W.F. Advisory boards: Takeda, Abbvie, MSD, Biogen, Janssen, Lilly, Speaker fees: Zambon, Takeda, Sandoz, Pfizer. Research grants: Pfizer, Ferring. A.I.; R.L.V.; C.A.; C.F.; G.S.; S.G.; E.G.; C.F.; M.S.; V.T.; D.C.; S.P.; S.R.; D.V.; M.E.D.S.; R.V.; M.G.M.; M.M.; no conflict of interest to declare.

## Supporting information


**Table S1:** Baseline characteristics according to previous exposure to Ustekinumab (A), anti‐TNFs (B), Vedolizumab (C), anti‐JAK (D). CRP: C‐reactive protein; SEM: standard error of the mean. Per protocol analysis.

**Table S2:** Outcomes according previous treatment with Ustekinumab (A), anti‐TNFs (B), vedolizumab (C) and small molecules (D). pMS = partial Mayo score; SFR = steroid free remission; Uste = Ustekinumab. Per protocol analysis.

**Table S3:** Outcomes according previous treatment with Ustekinumab (A) anti‐TNFs (B), vedolizumab (C) and small molecules (D), analyzed according to the intention‐to‐treat analysis (ITT). pMS = partial Mayo score; SFR = steroid free remission; Uste = Ustekinuma.

**Figure S1:** Treatment persistence at week 12 (A) and at week 24 (B).

## Data Availability

The data underlying this article will be shared on reasonable request to the corresponding author.

## References

[1] P. Rutgeerts , W. J. Sandborn , B. G. Feagan , et al., “Infliximab for Induction and Maintenance Therapy for Ulcerative Colitis,” New England Journal of Medicine 353, no. 23 (2005): 2462–2476, 10.1056/nejmoa050516. 16339095

[2] W. Reinisch , W. J. Sandborn , D. W. Hommes , et al., “Adalimumab for Induction of Clinical Remission in Moderately to Severely Active Ulcerative Colitis: Results of a Randomised Controlled Trial,” Gut 60, no. 6 (2011): 780–787, 10.1136/gut.2010.221127. 21209123

[3] W. J. Sandborn , B. G. Feagan , C. Marano , et al., “Subcutaneous Golimumab Induces Clinical Response and Remission in Patients With Moderate‐to‐Severe Ulcerative Colitis,” Gastroenterology 146, no. 1 (2014): 85–95, 10.1053/j.gastro.2013.05.048. 23735746

[4] B. G. Feagan , P. Rutgeerts , B. E. Sands , et al., “Vedolizumab as Induction and Maintenance Therapy for Ulcerative Colitis,” New England Journal of Medicine 369, no. 8 (2013): 699–710, 10.1056/nejmoa1215734. 23964932

[5] B. E. Sands , W. J. Sandborn , R. Panaccione , et al., “Ustekinumab as Induction and Maintenance Therapy for Ulcerative Colitis,” New England Journal of Medicine 381, no. 13 (2019): 1201–1214, 10.1056/nejmoa1900750. 31553833

[6] W. J. Sandborn , C. Su , B. E. Sands , et al., “Tofacitinib as Induction and Maintenance Therapy for Ulcerative Colitis,” New England Journal of Medicine 376, no. 18 (2017): 1723–1736, 10.1056/nejmoa1606910. 28467869

[7] B. G. Feagan , S. Danese , E. V. Loftus Jr , et al., “Filgotinib as Induction and Maintenance Therapy for Ulcerative Colitis (SELECTION): A Phase 2b/3 Randomised, Placebo‐Controlled Trial,” Lancet 397, no. 10292 (2021): 2372–2384, 10.1016/s0140-6736(21)00666-8. 34090625

[8] S. Danese , S. Vermeire , W. Zhou , et al., “Upadacitinib as Induction and Maintenance Therapy for Moderately to Severely Active Ulcerative Colitis,” Lancet 399, no. 10341 (2022): 2113–2128, 10.1016/s0140-6736(22)00581-5. 35644166

[9] W. J. Sandborn , B. G. Feagan , G. D'Haens , et al., “Ozanimod as Induction and Maintenance Therapy for Ulcerative Colitis,” New England Journal of Medicine 385, no. 14 (2021): 1280–1291, 10.1056/nejmoa2033617. 34587385

[10] W. J. Sandborn , S. Vermeire , L. Peyrin‐Biroulet , et al., “Etrasimod as Induction and Maintenance Therapy for Ulcerative Colitis (ELEVATE),” Lancet 401, no. 10383 (2023): 1159–1171, 10.1016/s0140-6736(23)00061-2. 36871574

[11] G. D'Haens , M. Dubinsky , T. Kobayashi , et al., “Mirikizumab as Induction and Maintenance Therapy for Ulcerative Colitis,” New England Journal of Medicine (2023). 10.1056/NEJMoa2207940 37379135

[12] M. E. Charlson , P. Pompei , K. L. Ales , and C. MacKenzie , “A New Method of Classifying Prognostic Comorbidity in Longitudinal Studies: Development and Validation,” Journal of Chronic Diseases 40, no. 5 (1987): 373–383, 10.1016/0021-9681(87)90171-8. 3558716

[13] M. C. Dubinsky , L. Newton , L. Delbecque , et al., “Exploring Disease Remission and Bowel Urgency Severity Among Adults With Moderate to Severe Ulcerative Colitis: A Qualitative Study,” Patient Related Outcome Measures 13 (2022): 287–300, 10.2147/prom.s378759. 36582542 PMC9793422

[14] M. C. Dubinsky , P. M. Irving , R. Panaccione , et al., “Development of the Urgency Numeric Rating Scale for Bowel Urgency,” Journal of Patient‐Reported Outcomes 6, no. 1 (2022): 31, 10.1186/s41687-022-00439-w. 35362902 PMC8975984

[15] J. D. Lewis , S. Chuai , L. Nessel , G. R. Lichtenstein , F. N. Aberra , and J. H. Ellenberg , “Use of the Noninvasive Components of the Mayo Score to Assess Clinical Response in Ulcerative Colitis,” Inflammatory Bowel Diseases 14, no. 12 (2008): 1660–1666, 10.1002/ibd.20520. 18623174 PMC2597552

[16] Gastroenterology and Hepatology (N.Y.) , “Efficacy and Safety of Mirikizumab as Induction Therapy in Patients With Moderately to Severely Active Ulcerative Colitis: Results From the Phase 3 LUCENT‐1 Study,” supplement, Gastroenterology and Hepatology 18, no. 4S1 (April 2022): 7–8. PMC9122067 35610995

[17] Y. Takagi , T. Sato , T. Nishiguchi , et al., “Real‐World Effectiveness and Safety of Mirikizumab Induction Therapy in Ulcerative Colitis,” Alimentary Pharmacology & Therapeutics 61, no. 12 (2025): 1923–1934, 10.1111/apt.70140. 40205711 PMC12107223

[18] T. Sawada , M. Nakamura , T. Yamamura , et al., “Efficacy of Mirikizumab in Patients With Prior Ustekinumab Exposure: A Case Series,” Inflammatory Bowel Diseases 31, no. 7 (2025): 2040–2041, 10.1093/ibd/izaf018. 40198002 PMC12235129

[19] J. St‐Pierre , D. Choi , E. Fear , et al., “Mirikizumab in the Treatment of Ulcerative Colitis: Real‐World Data From a Large Tertiary Center,” Digestive Diseases and Sciences 70, no. 5 (2025): 1864–1872, 10.1007/s10620-025-08950-y. 40048134

[20] A. Levartovsky , M. Lukáš , C. M. Abitbol , et al., “Real‐World Evidence of Mirikizumab in Ulcerative Colitis: Two‐Center Retrospective Cohort,” Therapeutic Advances in Gastroenterology 18 (2025): 17562848251392074, 10.1177/17562848251392074. 41230020 PMC12602953

[21] M. Murgiano , P. Balestrieri , E. Calabrese , et al., “Effectiveness and Safety of Mirikizumab in Ulcerative Colitis: Real‐World Data From the Latium Net,” BMJ Open Gastroenterology 13, no. 1 (2026): e002232, 10.1136/bmjgast-2025-002232. PMC13150890 42086294

[22] G. D'Haens , P. D. R. Higgins , L. Peyrin‐Biroulet , et al., “Extended Induction and Prognostic Indicators of Response to Mirikizumab in Ulcerative Colitis: LUCENT Trials,” Inflammatory Bowel Diseases 30 (2024): 2335–2346, 10.1093/ibd/izae004. 38271613 PMC11630349

[23] S. Vieujean , B. E. Sands , R. Panaccione , et al., “Comparison of FDA and EMA Guidance on Drug Development in Ulcerative Colitis,” Journal of Crohn's and Colitis 19, no. 7 (2025): jjaf111, 10.1093/ecco-jcc/jjaf111. PMC12280173 40692351

[24] P. Patel , P. Belesiotis , N. S. Rai , et al., “Meta‐Analysis: Improvement of Bowel Urgency With Advanced Therapies for IBD,” Alimentary Pharmacology & Therapeutics (2025). 10.1111/apt.70446 41185967

[25] G. B. Nigam , J. K. Limdi , S. Bate , S. Hamdy , and D. H. Vasant , “Fecal Urgency in Ulcerative Colitis: Impact on Quality of Life,” Clinical Gastroenterology and Hepatology 22, no. 8 (2024): 1731–1733, 10.1016/j.cgh.2023.12.019. 38151168

[26] M. Dubinsky , A. P. Bleakman , R. Panaccione , et al., “Bowel Urgency in Ulcerative Colitis: Current Perspectives and Future Directions,” American Journal of Gastroenterology 118, no. 11 (2023): 1940–1953, 10.14309/ajg.0000000000002404. 37436151 PMC10617668

