# Supplementary material for: Induction and Prolonged Induction With Mirikizumab in Ulcerative Colitis—A Prospective, Real‐World Study From the Sicilian Network for Inflammatory Bowel Disease (SN‐IBD)
Source: United European Gastroenterol J. 2026 Jun 20;14(5):e70244. doi: 10.1002/ueg2.70244 (PMC13282687; doi:10.1002/ueg2.70244)
Supplement: Supplementary file 1 — Table S1: Baseline characteristics according to previous exposure to Ustekinumab (A), anti‐TNFs (B), Vedolizumab (C), anti‐JAK (D). CRP: C‐reactive protein; SEM: standard error of the mean. Per protocol analysis. [file UEG2-14-e70244-s003.docx]

**A**

|  | **uste-experienced**  **n=36** | **uste-naïve**  **n=69** | **p** |
| --- | --- | --- | --- |
| Age; yrs mean ± SEM | 56 ± 2.55 | 48 ± 1.84 | **0.008** |
| Gender male (%) | 21/36 (58) | 39/69 (57) | 0.770 |
| Montreal UC; n (%)  E1; n (%)  E2; n (%)  E3; n (%) | 0  21/36 (58)  15/36 (42) | 5/69 (7)  39/69 (57)  25/69 (36) | 0.110  1  0.490 |
| IBD duration; yrs median range) | 7 (2-45) | 10 (1-36) | 0.456 |
| CCI; mean ± SEM | 1.05 ± 0.21 | 0.08 ± 0.16 | 0.479 |
| Partial Mayo score; median (range)  CRP positive at baseline | 4.5 (0-8)  13/36 (37) | 4 (0-6)  27/69 (39) | 0.382  0.843 |
| Steroids at baseline; n(%) | 21/69 (58) | 35/69 (51) | 0.565 |
| Failure to 1 advanced therapy n(%)  Failure to 2 advanced therapy n(%)  Failure to 3 advanced therapy n(%) | 0  8/36 (23)  28/36 (77) | 28/69 (39)  21/69 (30)  20/69 (29) | **0.005** |
| Extended induction; n(%) | 19/36 (53) | 33/69 (48) | 0.628 |

**B**

|  | **Anti-TNF experienced**  **n=89** | **Anti-TNF-naïve**  **n=16** | **p** |
| --- | --- | --- | --- |
| Age; yrs mean ± SEM | 48 ± 14.7 | 68± 8.9 | **< 0.001** |
| Gender male (%) | 49 (55) | 11 (69) | 0.29 |
| Montreal UC; n (%)  E1; n (%)  E2; n (%)  E3; n (%) | 5/89 (6)  52/89 (58)  32/89 (36) | 0/16 (0)  8/16 (50)  8/16 (50) | 0.31  0.55  0.29 |
| IBD duration; yrs median range) | 9.5 (1-45) | 7 (2-30) | 0.96 |
| CCI; mean ± SEM | 0.8 ± 1.3 | 1.4 ± 1.3 | 0.07 |
| Partial Mayo score; median (range) | 5/89 (0-9) | 6/16 (0-8) | 0.41 |
| CRP positive at baseline; n (%) | 31/89 (35) | 9/16 (56) | 0.11 |
| Steroids at baseline; n(%) | 51/89 (57) | 5/16 (31) | **0.05** |
| Failure to 1 advanced therapy n(%)  Failure to 2 advanced therapy n(%)  Failure to 3 advanced therapy n(%) | 25/89 (28)  21/89 (24)  45/89 (51) | 9/16 (56)  6/16 (38)  3/16 (19) | **0.01** |
| Extended induction; n(%) | 46/89 (52) | 11/16 (69) | 0.21 |

**C**

|  | **Vedolizumab experienced**  **n=45** | **Vedolizumab-naïve**  **n=60** | **p** |
| --- | --- | --- | --- |
| Age; yrs mean ± SEM | 56.5± 15.7 | 46.7± 14.4 | 0.06 |
| Gender male (%) | 28/45 (62) | 32/60 (53) | 0.35 |
| Montreal UC; n (%)  E1; n (%)  E2; n (%)  E3; n (%) | 2/45 (4)  23/45 (51)  20/45 (44) | 3/60 (5)  37/60 (62)  20/60 (33) | 0.80  0.26  0.25 |
| IBD duration; yrs median range) | 10 (2-45) | 8 (1-34) | 0.26 |
| CCI; mean ± SEM | 1.13 ± 1.6 | 0.74 ± 1.13 | 0.36 |
| Partial Mayo score; median (range)  CRP positive at baseline | 5 (0-8)  23/45 (51) | 6 (0-9)  17/60 (28) | 0.37  **0.01** |
| Steroids at baseline; n(%) | 10/45 (22) | 31/60 (52) | **0.001** |
| Failure to 1 advanced therapy n(%)  Failure to 2 advanced therapy n(%)  Failure to 3 advanced therapy n(%) | 3/45 (7)  12/45 (27)  30/45 (76) | 23/60 (38)  15/60 (25)  22/60 (37) | <**0.001** |
| Extended induction; n(%) | 20/45 (44) | 25/60 (42) | 0.83 |

**D**

|  | **Anti-JAK experienced**  **n=40** | **Anti-JAK**  **naïve**  **n=65** | **p** |
| --- | --- | --- | --- |
| Age; yrs mean ± SEM | 51.9± 15.1 | 50.5 ± 16.1 | 0.65 |
| Gender male (%) | 27/40 (67) | 33/65 (51) | 0.10 |
| Montreal UC; n (%)  E1; n (%)  E2; n (%)  E3; n (%) | 1/40 (2.5)  24/40 (60)  15/40 (37) | 4/65 (6)  37/65 (57)  25/65 (38) | 0.41  0.76  0.91 |
| IBD duration; yrs median (range) | 9.5 (1-45) | 9 (1-36) | 0.39 |
| CCI; mean ± SEM | 1.02 ± 1.5 | 0.8 ± 1.2 | 0.59 |
| Partial Mayo score; median (range)  CRP positive at baseline | 4 (0-9)  14/40 (35) | 6 (0-9)  26/65 (40) | 0.61 |
| Steroids at baseline; n(%) | 22/40 (55) | 35/65 (54) | 0.92 |
| Failure to 1 advanced therapy n(%)  Failure to 2 advanced therapy n(%)  Failure to 3 advanced therapy n(%) | 0  3/40 (7.5)  37/40 (92) | 28/65 (43)  24/65 (36)  15/65 (20) | <**0.001** |
| Extended induction; n(%) | 18/40 (45) | 40/65 (61) | 0.11 |

**Supplementary table 1**. Baseline characteristics according to previous exposure to Ustekinumab (A), anti-TNFs (B), Vedolizumab (C), anti-JAK (D). CRP: C-reactive protein; SEM: standard error of the mean. Per protocol analysis.
