# Supplementary material for: Induction and Prolonged Induction With Mirikizumab in Ulcerative Colitis—A Prospective, Real‐World Study From the Sicilian Network for Inflammatory Bowel Disease (SN‐IBD)
Source: United European Gastroenterol J. 2026 Jun 20;14(5):e70244. doi: 10.1002/ueg2.70244 (PMC13282687; doi:10.1002/ueg2.70244)
Supplement: Supplementary file 2 — Table S2: Outcomes according previous treatment with Ustekinumab (A), anti‐TNFs (B), vedolizumab (C) and small molecules (D). pMS = partial Mayo score; SFR = steroid free remission; Uste = Ustekinumab. Per protocol analysis. [file UEG2-14-e70244-s004.docx]

|  | **12 weeks** | | **p** | **24 weeks** | | **p** |
| --- | --- | --- | --- | --- | --- | --- |
| **Variable** | **Uste-experienced**  **n= 36** | **Uste-naïve**  **n= 69** |  | **Uste-experienced**  **n=32** | **Uste-naïve**  **n=39** |  |
| pMS; median (range) | 2 (0-9) | 1 (0-8) | 0.555 | 1 (0-2) | 0,5 (0-9) | 0.261 |
| Response; n(%) | 8/36 (22) | 18/69 (26) | 0.739 | 5/32 (16) | 9/39 (23) | 0.465 |
| SFR; n(%) | 16/36 (44) | 38/69 (55) | 0.387 | 21/32 (66) | 26/39 (67) | 0.929 |
| Urgency; median (range) | 3 (0-9) | 2 (0-7) | **0.047** | 2 (0-10) | 0 (0-6) | 0.072 |
| Treatment failure; n(%) | 2/36 (6) | 3/69 (4) | 0.726 | 3/32 (9) | 6/39 (15) | 0.763 |
| AEs; n(%) | 2/36 (5) | 2/69 (3) | 0.067 | 0/32 (0) | 1/39 (3) | 0.573 |

**A**

|  | **12 weeks** | | **p** | **24 weeks** | | **p** |
| --- | --- | --- | --- | --- | --- | --- |
| **Variable** | **Anti-TNF experienced**  **n= 89** | **Anti-TNF naïve**  **n= 16** |  | **Anti-TNF experienced**  **n= 59** | **Anti-TNF naïve**  **n=12** |  |
| pMS; median (range) | 2 (0-9) | 3 (0-6) | 0.51 | 1 (0-9) | 1 (0-6) | 0.89 |
| Response; n(%) | 22/89 (25) | 4/16 (25) | 1 | 12/59 (20) | 2/12 (17) | 0.81 |
| SFR; n(%) | 47/89 (53) | 9/16 (56) | 0.82 | 40/59 (68) | 7/12 (58) | 0.50 |
| Urgency; median (range) | 2 (0-9) | 3 (0-7) | 0.74 | 0 (0-10) | 0 (0-8) | 0.78 |
| Treatment failure; n(%) | 5/89 (6) | 0/16 | 0.36 | 6/59 (10) | 2/12 (17) | 0.48 |
| AEs; n(%) | 2/89 (2) | 2/16 (12) | **0.04** | 1/59 (2) | 0/12 | 0.62 |

**B**

**C**

|  | **12 weeks** | | **p** | **24 weeks** | | **p** |
| --- | --- | --- | --- | --- | --- | --- |
| **Variable** | **Vedolizumab experienced**  **n= 45** | **Vedolizumab naïve**  **n= 60** |  | **Vedolizumab experienced**  **n= 32** | **Vedolizumab naïve**  **n= 39** |  |
| pMS; median (range) | 3 (0-9) | 1 (0-8) | 0.051 | 1 (0-9) | 0.5 (0-4) | 0.23 |
| Response; n(%) | 12/45 (27) | 12/60 (20) | 0.48 | 7/32 (22) | 7/39 (18) | 0.77 |
| SFR; n(%) | 21/45 (47) | 35/60 (58) | 0.24 | 20/32 (62.5) | 27/39 (69) | 0.62 |
| Urgency; median (range) | 3 (0-9) | 1 (0-9) | **0.028** | 1.5 (0-10) | 0 (0-6) | 0.18 |
| Treatment failure; n(%) | 4/45 (9) | 4/60 (7) | 0.72 | 4 /32(12.5) | 2/39 (5) | 0.41 |
| AEs; n(%) | 3/45 (7) | 2/60 (3) | 0.65 | 0/32 (0) | 0/39 (0) | 1 |

**D**

|  | **12 weeks** | | **p** | **24 weeks** | | **p** |
| --- | --- | --- | --- | --- | --- | --- |
| **Variable** | **Anti-JAK experienced**  **n= 40** | **Anti-JAK naïve**  **n= 65** |  | **Anti-JAK experienced**  **n= 31** | **Anti-JAK naïve**  **n= 40** |  |
| pMS; median (range) | 2 (0-8) | 2 (0-8) | 0.68 | 1 (0-8) | 1.5 (0-9) | 0. 31 |
| Response; n(%) | 6/40 (15) | 18/65 (28) | 0.21 | 4/31 (13) | 10/40 (25) | 0.23 |
| SFR; n(%) | 23/40 (58) | 31/65 (48) | 0.19 | 23/31 (74) | 22/40 (55) | 0.12 |
| Urgency; median (range) | 2 (0-8) | 2 (0-7) | 0.50 | 0 (0-8) | 0.5 (0-10) | 0.95 |
| Treatment failure; n(%) | 4/40 (10) | 3/65 (5) | 0.26 | 2/31 (6) | 4/40 (10) | 0.69 |
| AEs; n(%) | 0/40 (0) | 1/65 (1.6) | 1.00 | 0/31 (0) | 1/40 (3) | 1.00 |

**Supplementary Table 2.** Outcomes according previous treatment with Ustekinumab (A), anti-TNFs (B), vedolizumab (C) and small molecules (D). pMS=partial Mayo score; SFR= steroid free remission; Uste= Ustekinumab. Per protocol analysis.
