# Supplementary figures and images for: Induction and Prolonged Induction With Mirikizumab in Ulcerative Colitis—A Prospective, Real‐World Study From the Sicilian Network for Inflammatory Bowel Disease (SN‐IBD)
Source: United European Gastroenterol J. 2026 Jun 20;14(5):e70244. doi: 10.1002/ueg2.70244 (PMC13282687; doi:10.1002/ueg2.70244)

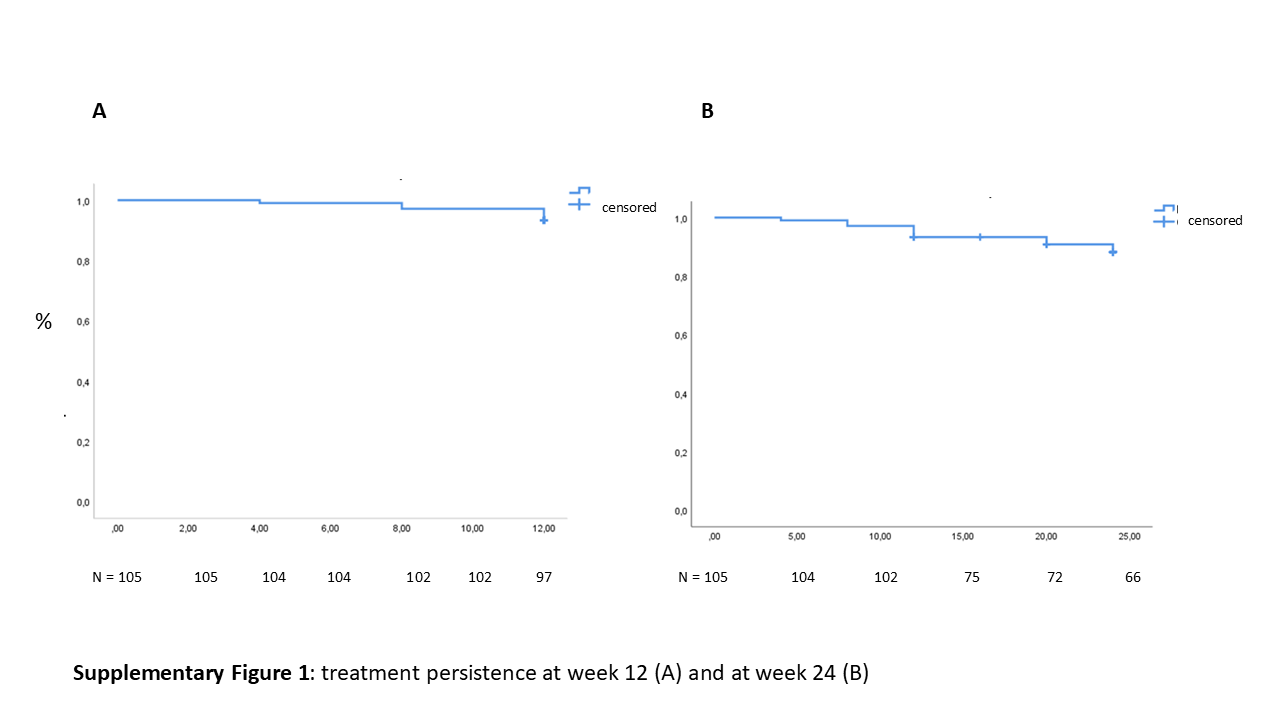

Supplement: Supplementary file 4 — Figure S1: Treatment persistence at week 12 (A) and at week 24 (B). [file UEG2-14-e70244-s002.tif]
